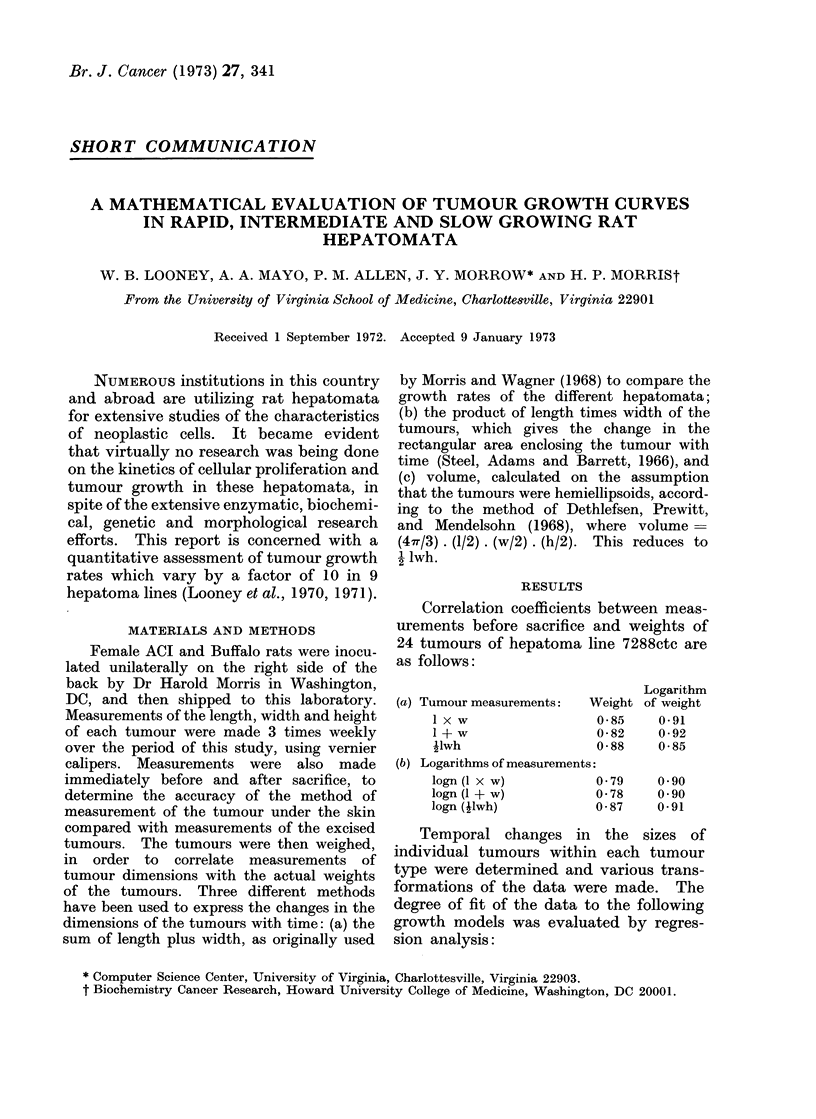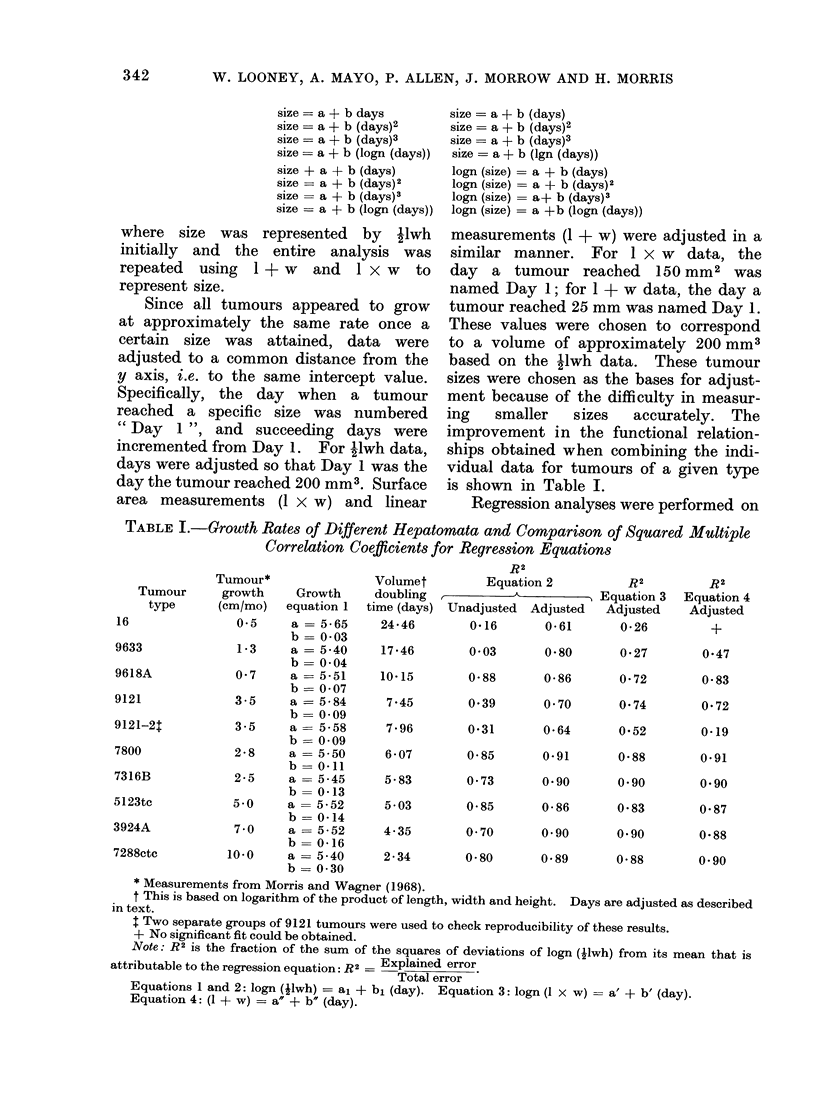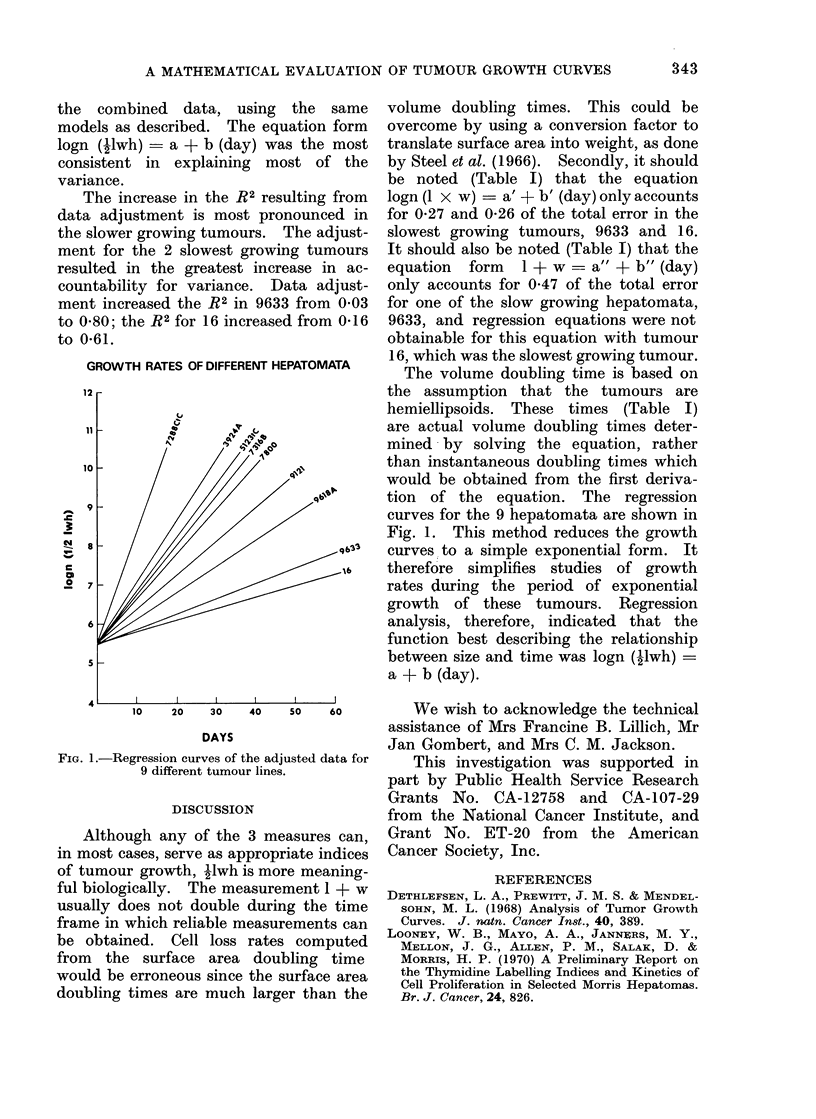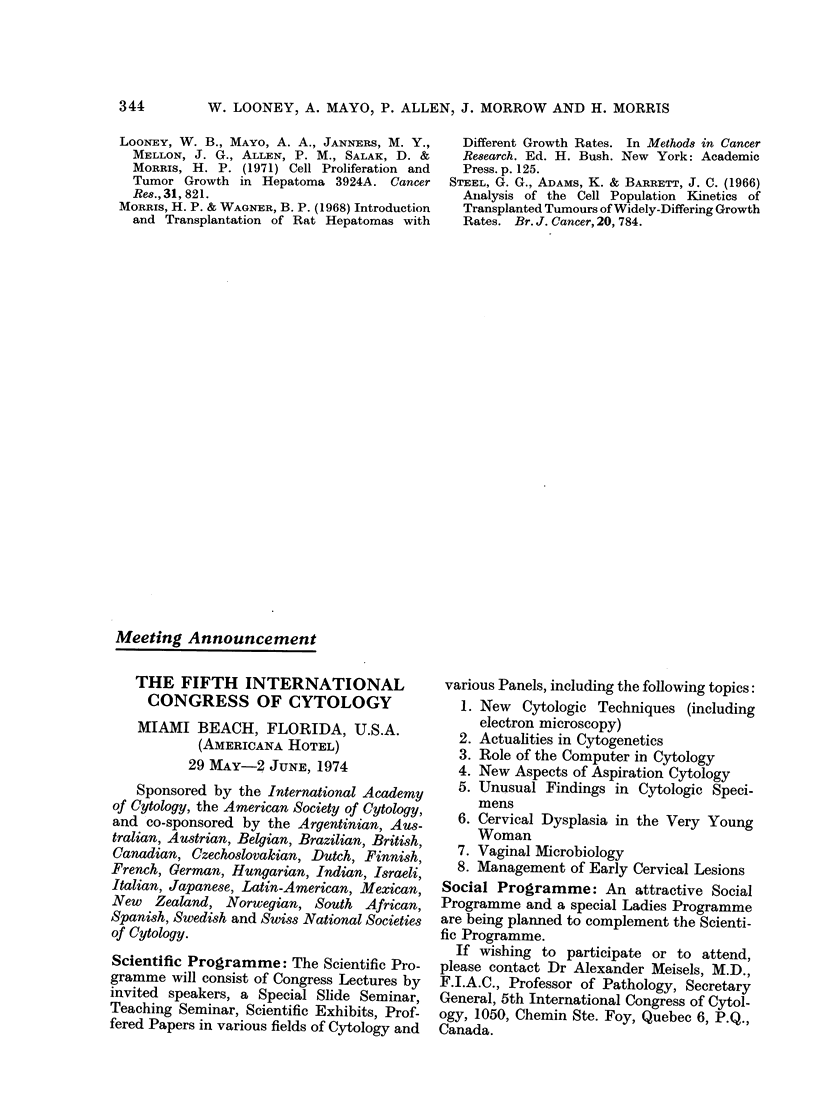# A mathematical evaluation of tumour growth curves in rapid, intermediate and slow growing rat hepatomata.

**DOI:** 10.1038/bjc.1973.41

**Published:** 1973-04

**Authors:** W. B. Looney, A. A. Mayo, P. M. Allen, J. Y. Morrow, H. P. Morris


*Br. J. Cancer* (1973) **27**, 341

### SHORT COMMUNICATION

### A MATHEMATICAL EVALUATION OF TUMOUR GROWTH CURVES IN RAPID, INTERMEDIATE AND SLOW GROWING RAT HEPATOMATA

W. B. LOONEY, A. A. MAYO, P. M. ALLEN, J. Y. MORROW* AND H. P. MORRIS†

*From the University of Virginia School of Medicine, Charlottesville, Virginia* 22901

Received 1 September 1972. Accepted 9 January 1973

NUMEROUS institutions in this country and abroad are utilizing rat hepatomata for extensive studies of the characteristics of neoplastic cells. It became evident that virtually no research was being done on the kinetics of cellular proliferation and tumour growth in these hepatomata, in spite of the extensive enzymatic, biochemical, genetic and morphological research efforts. This report is concerned with a quantitative assessment of tumour growth rates which vary by a factor of 10 in 9 hepatoma lines (Looney *et al.*, 1970, 1971).

### MATERIALS AND METHODS

Female ACI and Buffalo rats were inoculated unilaterally on the right side of the back by Dr Harold Morris in Washington, DC, and then shipped to this laboratory. Measurements of the length, width and height of each tumour were made 3 times weekly over the period of this study, using vernier calipers. Measurements were also made immediately before and after sacrifice, to determine the accuracy of the method of measurement of the tumour under the skin compared with measurements of the excised tumours. The tumours were then weighed, in order to correlate measurements of tumour dimensions with the actual weights of the tumours. Three different methods have been used to express the changes in the dimensions of the tumours with time: (a) the sum of length plus width, as originally used by Morris and Wagner (1968) to compare the growth rates of the different hepatomata; (b) the product of length times width of the tumours, which gives the change in the rectangular area enclosing the tumour with time (Steel, Adams and Barrett, 1966), and (c) volume, calculated on the assumption that the tumours were hemiellipsoids, according to the method of Dethlefsen, Prewitt, and Mendelsohn (1968), where volume = $(4\pi/3) . (l/2) . (w/2) . (h/2)$. This reduces to $\frac{1}{2}$ lwh.

### RESULTS

Correlation coefficients between measurements before sacrifice and weights of 24 tumours of hepatoma line 7288ctc are as follows:

| | Weight | Logarithm of weight |
|---|---|---|
| (*a*) Tumour measurements: | | |
| $l \times w$ | 0·85 | 0·91 |
| $l + w$ | 0·82 | 0·92 |
| $\frac{1}{2}$lwh | 0·88 | 0·85 |
| (*b*) Logarithms of measurements: | | |
| logn $(l \times w)$ | 0·79 | 0·90 |
| logn $(l + w)$ | 0·78 | 0·90 |
| logn ($\frac{1}{2}$lwh) | 0·87 | 0·91 |

Temporal changes in the sizes of individual tumours within each tumour type were determined and various transformations of the data were made. The degree of fit of the data to the following growth models was evaluated by regression analysis:

* Computer Science Center, University of Virginia, Charlottesville, Virginia 22903.
† Biochemistry Cancer Research, Howard University College of Medicine, Washington, DC 20001.

| | |
|---|---|
| size = a + b days | size = a + b (days) |
| size = a + b $(days)^2$ | size = a + b $(days)^2$ |
| size = a + b $(days)^3$ | size = a + b $(days)^3$ |
| size = a + b (logn (days)) | size = a + b (lgn (days)) |
| size + a + b (days) | logn (size) = a + b (days) |
| size = a + b $(days)^2$ | logn (size) = a + b $(days)^2$ |
| size = a + b $(days)^3$ | logn (size) = a+ b $(days)^3$ |
| size = a + b (logn (days)) | logn (size) = a +b (logn (days)) |

where size was represented by $\frac{1}{2}$lwh initially and the entire analysis was repeated using l + w and l × w to represent size.

Since all tumours appeared to grow at approximately the same rate once a certain size was attained, data were adjusted to a common distance from the *y* axis, *i.e.* to the same intercept value. Specifically, the day when a tumour reached a specific size was numbered " Day 1 ", and succeeding days were incremented from Day 1. For $\frac{1}{2}$lwh data, days were adjusted so that Day 1 was the day the tumour reached 200 $mm^3$. Surface area measurements (l × w) and linear measurements (l + w) were adjusted in a similar manner. For l × w data, the day a tumour reached 150 $mm^2$ was named Day 1; for l + w data, the day a tumour reached 25 mm was named Day 1. These values were chosen to correspond to a volume of approximately 200 $mm^3$ based on the $\frac{1}{2}$lwh data. These tumour sizes were chosen as the bases for adjustment because of the difficulty in measuring smaller sizes accurately. The improvement in the functional relationships obtained when combining the individual data for tumours of a given type is shown in Table I.

Regression analyses were performed on

TABLE I.—*Growth Rates of Different Hepatomata and Comparison of Squared Multiple Correlation Coefficients for Regression Equations*

| Tumour type | Tumour* growth (cm/mo) | Growth equation 1 | Volume† doubling time (days) | $R^2$ Equation 2 Unadjusted | $R^2$ Equation 2 Adjusted | $R^2$ Equation 3 Adjusted | $R^2$ Equation 4 Adjusted |
|---|---|---|---|---|---|---|---|
| 16 | 0·5 | a = 5·65<br>b = 0·03 | 24·46 | 0·16 | 0·61 | 0·26 | + |
| 9633 | 1·3 | a = 5·40<br>b = 0·04 | 17·46 | 0·03 | 0·80 | 0·27 | 0·47 |
| 9618A | 0·7 | a = 5·51<br>b = 0·07 | 10·15 | 0·88 | 0·86 | 0·72 | 0·83 |
| 9121 | 3·5 | a = 5·84<br>b = 0·09 | 7·45 | 0·39 | 0·70 | 0·74 | 0·72 |
| 9121–2‡ | 3·5 | a = 5·58<br>b = 0·09 | 7·96 | 0·31 | 0·64 | 0·52 | 0·19 |
| 7800 | 2·8 | a = 5·50<br>b = 0·11 | 6·07 | 0·85 | 0·91 | 0·88 | 0·91 |
| 7316B | 2·5 | a = 5·45<br>b = 0·13 | 5·83 | 0·73 | 0·90 | 0·90 | 0·90 |
| 5123tc | 5·0 | a = 5·52<br>b = 0·14 | 5·03 | 0·85 | 0·86 | 0·83 | 0·87 |
| 3924A | 7·0 | a = 5·52<br>b = 0·16 | 4·35 | 0·70 | 0·90 | 0·90 | 0·88 |
| 7288ctc | 10·0 | a = 5·40<br>b = 0·30 | 2·34 | 0·80 | 0·89 | 0·88 | 0·90 |

* Measurements from Morris and Wagner (1968).

† This is based on logarithm of the product of length, width and height. Days are adjusted as described in text.

‡ Two separate groups of 9121 tumours were used to check reproducibility of these results.

+ No significant fit could be obtained.

*Note:* $R^2$ is the fraction of the sum of the squares of deviations of logn ($\frac{1}{2}$lwh) from its mean that is attributable to the regression equation: $R^2 = \frac{\text{Explained error}}{\text{Total error}}$.

Equations 1 and 2: logn ($\frac{1}{2}$lwh) = $a_1 + b_1$ (day). Equation 3: logn (l × w) = a′ + b′ (day). Equation 4: (l + w) = a″ + b″ (day).

the combined data, using the same models as described. The equation form logn ($\frac{1}{2}$lwh) = a + b (day) was the most consistent in explaining most of the variance.

The increase in the $R^2$ resulting from data adjustment is most pronounced in the slower growing tumours. The adjustment for the 2 slowest growing tumours resulted in the greatest increase in accountability for variance. Data adjustment increased the $R^2$ in 9633 from 0·03 to 0·80; the $R^2$ for 16 increased from 0·16 to 0·61.

FIG. 1.—Regression curves of the adjusted data for 9 different tumour lines.

DISCUSSION

Although any of the 3 measures can, in most cases, serve as appropriate indices of tumour growth, $\frac{1}{2}$lwh is more meaningful biologically. The measurement l + w usually does not double during the time frame in which reliable measurements can be obtained. Cell loss rates computed from the surface area doubling time would be erroneous since the surface area doubling times are much larger than the volume doubling times. This could be overcome by using a conversion factor to translate surface area into weight, as done by Steel *et al.* (1966). Secondly, it should be noted (Table I) that the equation logn (l × w) = a′ + b′ (day) only accounts for 0·27 and 0·26 of the total error in the slowest growing tumours, 9633 and 16. It should also be noted (Table I) that the equation form l + w = a″ + b″ (day) only accounts for 0·47 of the total error for one of the slow growing hepatomata, 9633, and regression equations were not obtainable for this equation with tumour 16, which was the slowest growing tumour.

The volume doubling time is based on the assumption that the tumours are hemiellipsoids. These times (Table I) are actual volume doubling times determined by solving the equation, rather than instantaneous doubling times which would be obtained from the first derivation of the equation. The regression curves for the 9 hepatomata are shown in Fig. 1. This method reduces the growth curves to a simple exponential form. It therefore simplifies studies of growth rates during the period of exponential growth of these tumours. Regression analysis, therefore, indicated that the function best describing the relationship between size and time was logn ($\frac{1}{2}$lwh) = a + b (day).

We wish to acknowledge the technical assistance of Mrs Francine B. Lillich, Mr Jan Gombert, and Mrs C. M. Jackson.

This investigation was supported in part by Public Health Service Research Grants No. CA-12758 and CA-107-29 from the National Cancer Institute, and Grant No. ET-20 from the American Cancer Society, Inc.

REFERENCES

DETHLEFSEN, L. A., PREWITT, J. M. S. & MENDELSOHN, M. L. (1968) Analysis of Tumor Growth Curves. *J. natn. Cancer Inst.*, **40**, 389.

LOONEY, W. B., MAYO, A. A., JANNERS, M. Y., MELLON, J. G., ALLEN, P. M., SALAK, D. & MORRIS, H. P. (1970) A Preliminary Report on the Thymidine Labelling Indices and Kinetics of Cell Proliferation in Selected Morris Hepatomas. *Br. J. Cancer*, **24**, 826.

LOONEY, W. B., MAYO, A. A., JANNERS, M. Y., MELLON, J. G., ALLEN, P. M., SALAK, D. & MORRIS, H. P. (1971) Cell Proliferation and Tumor Growth in Hepatoma 3924A. *Cancer Res.*, **31**, 821.

MORRIS, H. P. & WAGNER, B. P. (1968) Introduction and Transplantation of Rat Hepatomas with Different Growth Rates. In *Methods in Cancer Research*. Ed. H. Bush. New York: Academic Press. p. 125.

STEEL, G. G., ADAMS, K. & BARRETT, J. C. (1966) Analysis of the Cell Population Kinetics of Transplanted Tumours of Widely-Differing Growth Rates. *Br. J. Cancer*, **20**, 784.